# The Paradigm Shift in Clinical Stage II Non-Small-Cell Lung Cancer Management: A Comprehensive Review of Optimal Surgical and Systemic Approaches

**DOI:** 10.3390/cancers18111680

**Published:** 2026-05-22

**Authors:** Tyler W. Wilson, Jessica S. Donington

**Affiliations:** 1 Department of Surgery, University of Chicago Medicine, Chicago, IL 60637, USA; 2 Section of Thoracic Surgery, University of Chicago Medicine, Chicago, IL 60637, USA; jdonington@bsd.uchicago.edu

**Keywords:** non-small cell lung cancer, neoadjuvant therapy, adjuvant therapy, perioperative therapy, immunotherapy, targeted therapy

## Abstract

Lung cancer is one of the most common cancers worldwide, with non-small-cell lung cancer (NSCLC) being the most prevalent type. While surgical resection followed by adjuvant platinum-based chemotherapy has been the standard for curative-intent therapy for clinical stage II NSCLC since 2005, disappointing 5-year survival prompted the exploration of newer systemic therapies. In recent years, several landmark trials increasingly support the use of immunotherapy and molecular targeted treatments. The evidence for neoadjuvant chemoimmunotherapy is exciting, but the transition from a surgery-first approach to a new standard of care carries important challenges, including increased surgical attrition, intraoperative technical difficulty, and delays in care. This article provides a comprehensive review of the optimal treatments and emerging therapies for resectable stage II NSCLC. By systematically analyzing recent advances and challenges in NSCLC treatment strategies, we aim to highlight a paradigm shift toward a more molecularly guided, individualized treatment sequence in stage II NSCLC care, with the goal of maximizing each patient’s curative potential.

## Simple Summary

Surgery has traditionally been the first step and cornerstone of treatment for stage II lung cancer. In recent years, several influential trials have shifted the standard of care toward a biomarker-driven, multidisciplinary approach integrating systemic and surgical management for these patients. This review summarizes the latest evidence supporting this shift, including published results on immunotherapy and targeted therapy and their implications for surgery. Although these studies have already transformed the current approach to stage II disease management, ongoing phase II and III trials—some with a novel surgical focus—will continue to improve our understanding of the optimal systemic and surgical treatments for stage II disease.

## 1. Introduction

Lung cancer remains among the most common malignancies worldwide, making up about 11% of all new cancer cases and over 20% of cancer-related deaths annually [1]. Non-small-cell lung cancer (NSCLC) comprises roughly 80–85% of all lung cancer cases and represents most of the disease burden. Stage II disease—defined as T1-2N1, T1N2a, or T3N0 lesions—accounts for 7% to 10% of new NSCLC diagnoses [2]. For decades, surgical resection has been the cornerstone of curative-intent therapy for NSCLC. Although surgery offers the highest probability of long-term survival, the risk of recurrence is substantial. In stage II disease, where surgery was historically the only treatment, five-year survival rates range from 36% to 46% [3].

To address these suboptimal outcomes, the treatment landscape evolved to include adjuvant systemic therapy. Landmark trials, including the International Adjuvant Lung Cancer Trial and the JBR.10 Trial established adjuvant platinum-based doublet therapy as the standard of care for resected stage II NSCLC [3,4]. These and other trials demonstrated that adding adjuvant platinum-based chemotherapy conferred an absolute 5% to 15% five-year survival benefit in resected stage II patients [4,5,6,7]. Despite this advancement, the “one-size-fits-all” approach plateaued, with >50% of patients experiencing disease relapse.

In recent years, the management of stage II NSCLC has entered a transformative era. Stage II disease is resectable but has a high risk of recurrence, and therefore, it is ideal for the integration of precision medicine and immunotherapy into the perioperative setting [8,9]. Previously, these systemic agents were reserved exclusively for the management of metastatic disease, while early-stage patients were limited to surgical resection and platinum-doublet therapy. This paradigm is shifting, as recent landmark clinical trials demonstrate that using more efficacious agents can increase pathologic response, better target micrometastatic disease, and improve long-term survival. Importantly, most pivotal trials enrolled mixed stage II-III populations and were not powered for stage-specific analysis; stage II conclusions therefore rely primarily on subgroup data and meta-analyses.

In this review, we aim to provide a comprehensive overview of the most recent practice-changing research and highlight the ongoing paradigm shift from a one-size-fits-all, surgery-first, and adjuvant chemotherapy approach to a personalized, molecular-profile and immune-biomarker-directed approach for stage II disease.

## 2. Methods

A non-systematic literature search was conducted on PubMed and the reference lists of key articles, using terms such as “stage II NSCLC,” “perioperative therapy,” “node-positive resectable lung cancer,” “targeted therapy,” “neoadjuvant therapy,” and “immunotherapy.” Abstracts were reviewed by both authors and selected based on their clinical significance and methodological rigor, with preference to prospective phase II/III randomized trials including stage II-specific patients, subsequent publications from these trials, and systematic meta-analyses. Only studies published since 2018 were included to focus on the integration of novel biomarkers and treatments. The evidence was qualitatively synthesized to evaluate current treatment paradigms, ongoing controversies in multimodal therapy, challenges in treatment transition, and future directions for personalized thoracic surgical oncology.

## 3. Immune Checkpoint Inhibitors

For stage II NSCLC, the standard of care has long been surgical resection followed by adjuvant platinum-doublet chemotherapy, which provides a modest 5% absolute improvement in overall survival (OS) [4]. However, since 2020, the integration of immune checkpoint inhibitors (ICIs) into treatment for locally advanced NSCLC has fundamentally altered this standard. Checkpoint inhibitors target regulatory proteins, such as the programmed cell death ligand 1 (PD-L1) or programmed cell death protein 1 (PD-1), to reset the host immune system to recognize and destroy malignant cells. This evolution began with the incorporation of adjuvant immunotherapy (IO), followed by a rapid expansion into the neoadjuvant and perioperative spaces. Several trials have evaluated the integration of IO into NSCLC treatment, with practice-changing results (Table 1) [10,11,12,13,14,15,16,17,18,19,20,21,22,23,24,25,26,27,28,29,30,31,32,33,34]. Notably, although these trials include stage II patients, stage II-specific conclusions, particularly in studies without dedicated subgroup analyses, are somewhat limited by a disproportionate contribution from stage III patients, especially in the neoadjuvant and perioperative trials.

### 3.1. Adjuvant Immunotherapy

Adjuvant IO is typically given after completing four cycles of platinum-based chemotherapy. The clinical validation of this approach comes from two phase III trials, IMpower010 and KEYNOTE-091, which provided strong evidence supporting the inclusion of atezolizumab and pembrolizumab in standard postoperative treatment protocols [10,13].

The IMpower010 trial was the first to show a significant clinical benefit for adjuvant IO. Resected stage IB to IIIA NSCLC patients were randomized to receive up to one year of atezolizumab or best supportive care after a mandatory course of adjuvant chemotherapy. The primary analysis revealed a statistically significant disease-free survival (DFS) benefit for stage II-IIIA patients with a PD-L1 expression ≥ 1% (HR 0.66) [10]. This benefit was especially notable in the high-PD-L1 group (≥50%), where the hazard ratio (HR) across all stages was striking, at 0.43. Fifty-three percent of patients enrolled in this trial had resected stage II disease. Recent five-year follow-up data further support this strategy, indicating improved overall survival (OS) in the high-expression subgroup, though the benefit in the PD-L1 1–49% range remains more modest [11,12].

The KEYNOTE-091 trial (also known as PEARLS) assessed the efficacy of adjuvant pembrolizumab in a similar patient population. Notably, adjuvant chemotherapy was recommended, but not mandatory; nevertheless, 86% of participants received it [13]. Pembrolizumab showed a significant improvement in DFS across the entire intention-to-treat population regardless of PD-L1 expression levels, with a median DFS of 53.6 months compared to 42.0 months in the placebo group [13]. Surprisingly, the significant survival benefit for patients with high-PD-L1-expressing tumors in the IMpower010 trial was not seen in this trial. Although the effect size in pure stage II subgroups remains less robust, this study led to the approval of pembrolizumab as adjuvant treatment in stage IB to IIIA, regardless of PD-L1 status, for resected patients who completed prior chemotherapy.

Taken together, these trials firmly establish adjuvant IO as a new standard-of-care option following resection and platinum-based chemotherapy for stage II NSCLC without actionable mutations. Atezolizumab provides the greatest benefit in patients with high PD-L1 expression, while pembrolizumab offers meaningful DFS improvement across PD-L1 strata, extending eligibility to a broader population.

### 3.2. Neoadjuvant Immunotherapy

The neoadjuvant approach to IO takes advantage of having the primary tumor in place to optimally prime immune cells against a broad array of tumor antigens and to achieve a deep pathological response prior to surgery, which is increasingly recognized as a surrogate for long-term survival [20,35]. Initial safety of IO in the neoadjuvant setting was demonstrated in CheckMate 159, a single-arm pilot trial including 21 patients with resectable stage I-IIIA NSCLC from Memorial Slone Kettering Cancer Center and Johns Hopkins University [14]. Patients received two doses of nivolumab, and 20/21 underwent resection without increased morbidity or mortality. There were no delays to resection, 95% were R0, and the major pathologic response (MPR) was 45% [15]. Nearly half (48%) of patients in this pilot had stage II disease. The LCMC3 study was a larger single-arm, multi-institutional phase II trial in which patients received two cycles of neoadjuvant atezolizumab monotherapy followed by surgical resection and optional adjuvant treatment. In this cohort, 43% of patients were downstaged and 92% of operations resulted in an R0 resection, further supporting the concept that neoadjuvant IO did not excessively delay or complicate curative-intent surgery [17,18].

The ground-breaking CheckMate 816 trial reported its initial results in 2022. This was a phase III trial comparing neoadjuvant chemotherapy with IO (chemo-IO) versus chemotherapy alone in patients with resectable stage IB (≥4 cm) to IIIA NSCLC. The addition of nivolumab significantly improved pathologic complete response (pCR) (24% vs. 2%), 5-year event-free survival (EFS) (49% vs. 34%), and 5-year OS (65% vs. 55%) [19,20]. These results challenged the long-standing surgery-first treatment paradigm for stage II disease, established neoadjuvant chemo-IO as a standard option, and led to the first FDA approval of this approach. This trial also ushered in the recognition of the strong association between pCR following induction therapy and survival in NSCLC, supporting tumor response as a key biomarker to further guide and personalize subsequent treatment decisions [20].

Across these trials, consistent downstaging, high R0 rates, and the absence of excess surgical morbidity supported neoadjuvant chemo-IO as a transformative strategy for resectable NSCLC, including stage II disease, and established it as a pillar of modern multimodality care.

### 3.3. Perioperative Immunotherapy

Building on findings from neoadjuvant trials, large phase III studies have also evaluated neoadjuvant chemo-IO followed by adjuvant single-agent IO, effectively maintaining the anti-tumor response and eliminating remaining dormant tumor cells after resection. This expansion of ICIs into the perioperative setting is further transforming systemic therapy for resectable stage II disease.

The KEYNOTE-671, AEGEAN, CheckMate 77T, and Neotorch trials collectively show that this “IO-sandwich” approach in stage II disease significantly increases the likelihood of achieving a pCR [21,25,27,28,34]. Similar to CheckMate816, adding IO to neoadjuvant chemotherapy increased pCR rates from 5% to 17–25%. More importantly, these trials show a marked improvement in EFS, with KEYNOTE-671 reporting the first statistically significant OS benefit with an HR of 0.72 and a 36-month survival rate of 71.3% compared to 64% in the control arm [21,25,27,28,34].

An equally important finding across these studies is that preoperative IO does not appear to compromise surgical safety or feasibility. In fact, many patients experienced tumor and/or nodal downstaging, which numerically allowed for less extensive and more minimally invasive resections [26,36]. Although these benefits are most pronounced in patients with high PD-L1 expression, data from AEGEAN and CheckMate 77T indicate that patients can benefit clinically regardless of PD-L1 expression [25,27].

Moving forward, perioperative trials are expanding the boundaries of current standards by testing next-generation IO agents and targeted therapy combinations. The NeoCOAST and NeoCOAST-2 trials are combining perioperative durvalumab with novel antibody–drug conjugates (ADCs) and achieving higher pathologic response rates [29,30]. Specifically, the addition of TROP2-directed ADCs, such as Datopotamab Deruxtecan (Dato-DXd), in NeoCOAST-2 resulted in a pCR rate of 35% and an MPR rate of 63% [30]. These intensified regimens demonstrate the potential for even greater efficacy across all PD-L1 subgroups, signaling a shift toward more personalized perioperative care that could further reduce recurrence and improve survival for stage II patients.

### 3.4. Evidence in Stage II-Specific Subgroups

Although landmark clinical trials have established the benefit of IO in resectable stage II-III NSCLC, specific trial-level data for the stage II subgroup remain limited, as stage II patients are only a subset of study populations and trials were not powered for stage-specific comparisons (Table 2). Importantly, the stage II subgroup analyses from CheckMate 816 and AEGEAN were not published directly. In these trials, the stage II EFSs were numerically inferior to that of the overall trial population (0.87 and 0.76 vs. 0.63 and 0.68, respectively) and crossed 1.0, failing to establish a statistically significant benefit, and the absolute number of stage II patients was small, thus limiting the statistical power of the subgroup [19,20,25]. Nonetheless, dedicated stage II analyses from two perioperative trials have demonstrated meaningful benefit. A subgroup analysis of the 239 stage II participants in KEYNOTE-671 confirmed that perioperative pembrolizumab significantly improved EFS, MPR, and pCR, with favorable trends in OS [24]. These results were consistent with those of the overall trial population. Similarly, the RATIONALE-315 trial conducted in China using tislelizumb, a PD-1 inhibitor, enrolled a higher proportion of stage II patients than other global studies and reported a particularly robust stage II EFS benefit at the interim analysis (HR 0.47; 95% CI 0.26–0.87), numerically superior to that in the stage IIIA subgroup [32]. At the final analysis, the overall population demonstrated a significant OS benefit, with a 36-month OS rate of 79.3% versus 69.3% in favor of IO [33].

These findings are corroborated by pooled analyses of neoadjuvant and perioperative trials. In a meta-analysis of eight randomized trials, Sorin et al. demonstrated a statistically significant EFS benefit with neoadjuvant chemo-IO in stage II disease, alongside improvements in OS, MPR, and pCR in the pooled stage II population [37]. Most recently, Rossi et al. provided a critical distinction between IO approaches, showing that neoadjuvant/perioperative chemo-IO demonstrated a significant EFS benefit in stage II patients, whereas adjuvant IO alone did not reach statistical significance [38].

**Table 2:** Stage II-specific outcomes from key immunotherapy and targeted therapy trials.

Trial Name	Percent Stage II	Disease-Free/Event-Free Survival	Overall Survival	Other Outcomes
IMpower010 [10,11,12]	53%	3Y IIA HR 0.68 (0.46–1.00) 3Y IIB HR 0.88 (0.54–1.18)	NR	NR
PEARLS/KEYNOTE-091 [13]	56%	2Y HR 0.70 (0.55–0.91)	NR	NR
CheckMate 159 [14,15,16]	48%	NR	NR	100% R0 Resection
LCMC3 [17,18]	39%	NR	NR	NR
CheckMate 816 [19,20]	35%	NR	NR	NR
KEYNOTE-671 [13,21,22,23,24,34]	30%	2Y HR 0.50 (0.34–0.74)	2Y HR 0.69 (0.43–1.11)	MPR 36.4% pCR 24.6% 94.9% R0 Resection
AEGEAN [25,26]	29%	1Y HR 0.76 (0.43–1.34)	NR	pCR 21.2%
CheckMate 77T [27]	35%	1Y HR 0.81 (0.46–1.43)	NR	pCR 29.6%
NEOTORCH [28]	19%	NR	NR	NR
NeoCOAST [29]	51%	NR	NR	NR
NeoCOAST-2 [30]	31%	NR	NR	NR
MDT-Bridge [31]	~20–25%	NR	NR	NR
RATIONALE-315 [32,33]	41%	4Y HR 0.55 (0.32–0.94)	4Y HR 0.58 (0.31–1.06)	MPR 59.1% pCR 38.7%
ADAURA [39,40,41,42]	34%	4Y HR 0.23 (0.18–0.30)	5Y HR 0.63 (0.34–1.12)	NR
NeoADAURA [43]	51%	NR	NR	MPR 22–27%
ALINA [44]	36%	NR	NR	NR
ELEVATE [45]	30%	NR	NR	NR
ALNEO [46,47]	~10–15%	NR	NR	NR
NAUTIKA1 [48]	25%	NR	NR	NR

Abbreviations: HR, hazard ratio; Y, year; NR, not reported; MPR, major pathologic response; pCR, pathologic complete response. Hazard ratios are expressed as HRs (95% confidence interval).

### 3.5. Sequencing Therapies

The integration of IO into the treatment of NSCLC has fundamentally redefined management strategies. As of 2026, the clinical debate has shifted from whether to use IOs or not to when to administer them, with clinicians now navigating three main approaches:

1. The neoadjuvant approach (CheckMate 816);
2. The adjuvant-only approach (IMPower 010, KEYNOTE-091);
3. The perioperative “sandwich” model (KEYNOTE-671, AEGEAN, CheckMate 77T).

The strongest evidence to date supports IO combined with platinum doublets as neoadjuvant or perioperative therapy. Across multiple randomized trials, HRs for EFS and DFS consistently favor the addition of IO in the neoadjuvant or perioperative setting, while the benefit of adjuvant administration is less consistent across trials and in more selective populations [12,19,22,25,27,49]. The mechanism for the improved outcomes is believed to be the enhanced priming of immune cells with the primary tumor in place to better target and eradicate micrometastatic disease. Preclinical data support larger, more diverse populations of circulating tumor-directed T-cells with neoadjuvant IO delivery compared with adjuvant IO approaches [14].

Importantly, no prospective head-to-head comparison of preoperative vs. postoperative IO in NSCLC has been reported to date, but clinical data from other tumor types strongly support this strategy. SWOG 1801 was a prospective, randomized trial comparing adjuvant versus perioperative pembrolizumab in resectable melanoma; the 24-month event-free survival HR was 0.58, favoring the periadjuvant approach [50]. The ongoing PROSPECT-Lung trial (NCT06632327) sponsored by the Alliance addresses this same question in NSCLC [51]. This pragmatic phase III trial aims to enroll 1100 patients with resectable stage IIA-IIIB disease without known driver mutations and directly compare the perioperative and adjuvant chemo-IO approaches. While survival outcomes will take years to mature, the preliminary data on surgical attrition, outcomes, and early pCR rates are highly anticipated.

It is important to note that the major adjuvant trials (e.g., IMpower010 and KEYNOTE-091) began before reports from the biomarker-directed perioperative trials were published and, therefore, included significant numbers of patients with EGFR and ALK mutations. Results are now reported with and without these patients, with the differences in outcomes highlighting that integrating IO into the adjuvant setting requires careful biomarker testing to ensure the right patient populations receive the most effective post-surgical treatment.

**Figure 1:**
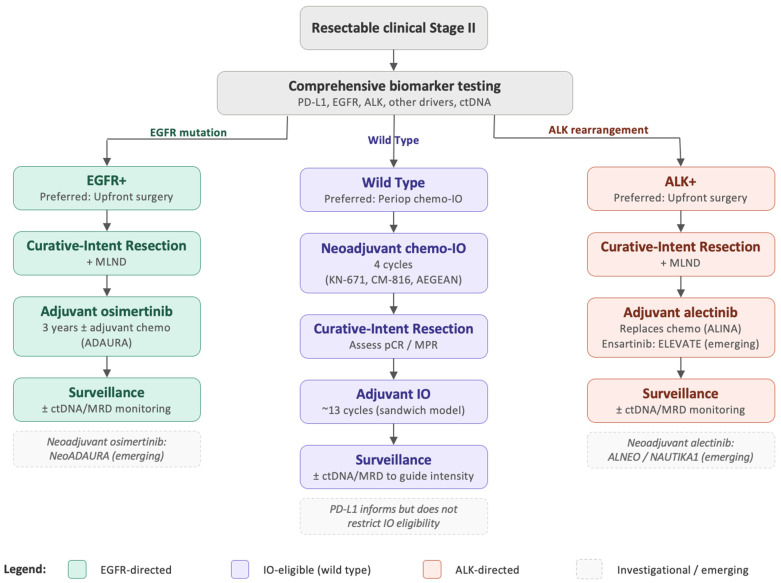
Stage II non-small-cell lung cancer treatment sequencing by biomarker. Abbreviations: PD-L1, programmed-death ligand 1; EGFR, epidermal growth factor receptor; ALK, anaplastic lymphoma kinase; ctDNA, circulating tumor DNA; MLND, mediastinal lymph node dissection; MRD, molecular residual disease; IO, immunotherapy; KN, KEYNOTE; CM, CheckMate; pCR, pathologic complete response; MPR, major pathologic response.

Overall, determining the ideal IO and surgery sequence for a specific patient requires a nuanced evaluation of tumor burden, PD-L1 expression, and, critically, the presence of actionable driver mutations (Figure 1). These observations highlight that IO-based strategies, while transformative, are only one part of a broader personalized treatment landscape. Currently, individual trial data and pooled meta-analyses support the addition of chemo-IO to the care of resectable stage II NSCLC. Efficacy data are most consistent for neoadjuvant or perioperative chemo-IO approaches with EFS HRs ranging from 0.47 to 0.71 across analyses [24,32,37,38]. The evidence for benefit with adjuvant chemo-IO is less robust; therefore, neoadjuvant and periadjuvant approaches should be strongly considered for resectable stage II disease while we await prospective head-to-head data.

## 4. Targeted Therapies

While chemo-IO has become the new standard for treating many patients with resectable stage II disease, targeted therapy provides a precision medicine approach for select subgroups of patients whose tumors contain specific oncogenic driver mutations. More than 20 such mutations currently have a targeted therapy approved for metastatic NSCLC. Recent landmark data have established a critical role for adjuvant tyrosine kinase inhibitors (TKIs) in improving survival among patients with early-stage disease and driver mutations, particularly EGFR mutations (Table 3) [39,40,41,42,43,44,45,46,47,48]. Patients with targetable mutations have a superior response to their targeted drugs than to chemo-IO, and have potential for increased toxicity when exposed to IO prior to TKIs [52]. It is therefore essential to determine the tumor’s molecular profile prior to initiating treatment. As such, the integration of comprehensive molecular profiling at the time of diagnosis has become essential to optimizing outcomes and long-term survivorship in mutation-driven stage II disease.

### 4.1. Epidermal Growth Factor Receptor (EGFR)-Targeted Therapy

Similar to the introduction of IO in resectable disease, EGFR-directed therapy in early-stage disease was initially evaluated in the adjuvant setting. The phase III ADAURA trial established osimertinib as the standard adjuvant therapy after complete resection of stage IB-IIIA EGFR-mutant NSCLC, with or without chemotherapy, demonstrating an 83% reduction in the risk of recurrence or death from disease [39]. Longitudinal analyses confirmed that this benefit translates into significant DFS and OS improvements compared with placebo (85% 5-year OS) [40,41]. Stage II patients derived substantial benefit, with deep reductions in recurrence risk, especially in the central nervous system—a historical site of frequent failure in EGFR-driven disease. These data support the use of prolonged (3-year) adjuvant TKI as part of multimodality care in this subgroup [41]. These data have shifted the expectations for adjuvant chemotherapy alone in this subgroup, which offers only a modest benefit in stage II disease.

Building on the success of adjuvant osimertinib, the NeoADAURA trial focuses on the neoadjuvant setting. This trial is assessing the efficacy of osimertinib with or without platinum-based chemotherapy versus chemotherapy alone in resectable stage II-IIIB EGFR-mutant NSCLC. Preliminary results showed a significant increase in MPR with TKI compared to chemotherapy alone, with no associated effect on surgical attrition or perioperative morbidity [43]. The mechanisms of action and tumor biology of TKIs differ significantly from those of chemotherapy or IO, yet early responses and safety signals are encouraging.

### 4.2. Anaplastic Lymphoma Kinase (ALK)-Targeted Kinase Inhibitors

The management of ALK-rearranged resectable stage II NSCLC has also seen a rapid pivot toward the integration of targeted therapy. The phase III ALINA trial showed that adjuvant alectinib significantly improves DFS compared with platinum-based chemotherapy in completely resected ALK-positive stage IB-IIIA NSCLC [44]. This established a new postoperative standard for this molecular subset of patients. Stage II patients in ALINA experienced a 65% relative reduction in recurrence risk and a 4-year DFS of 75%, compared with 46% in those receiving chemotherapy [44]. These findings paralleled the magnitude observed with ADAURA in EGFR-mutant disease, further highlighting the importance of comprehensive molecular profiling in all resectable stage II tumors.

Several additional perioperative ALK-TKI trials are currently underway (Table 3). The ELEVATE trial (ensartinib) evaluates adjuvant ALK-TKI and the ALNEO trial evaluates neoadjuvant followed by extended adjuvant alectinib in resectable stage III ALK-positive NSCLC, whereas NAUTIKA1 tests multiple target agents (including ALK inhibition) [45,47,48]. Although the data are immature, these trials are expected to clarify whether preoperative ALK inhibition can downstage disease, increase MPR/pCR, and further improve survival in resectable ALK-driven NSCLC.

These targeted-therapy trials have established that oncogene-directed treatment is not confined to metastatic NSCLC. Recent reviews and society consensus statements highlight that adjuvant osimertinib and alectinib are practice-changing for EGFR- and ALK-driven resected NSCLC, respectively, and that perioperative TKIs (NeoADAURA, ALNEO, NAUTIKA1) have the potential to alter the timing and sequencing of surgery in stages II–III [53,54,55]. Despite this progress, important challenges remain for stage II disease: assessing the benefit of neoadjuvant versus strictly adjuvant TKI, identifying the optimal treatment duration, and combining chemotherapy, radiotherapy, and targeted agents without excessive toxicity [56]. Current evidence supports molecular testing for all resectable stage II NSCLC and adjuvant use of targeted therapy in EGFR-mutant and ALK-positive cases.

### 4.3. Circulating Tumor DNA and Minimal Residual Disease

Circulating tumor DNA (ctDNA) and molecular residual disease (MRD) detection represent emerging, potentially transformative biomarker-focused prognostic tools that complement static tissue biomarker testing in resectable NSCLC. Postoperative ctDNA positivity can identify patients with persistent micrometastatic disease at increased risk of recurrence, while ctDNA or MRD clearance may ultimately support de-escalation of adjuvant therapy—a question particularly relevant for stage II patients who achieve pCR following neoadjuvant chemo-IO. The strongest support for this method of surveillance comes from the ADAURA MRD sub-study, in which MRD clearance on adjuvant osimertinib correlated with improved DFS in EGFR-mutant early-stage disease [42]. Correlative data are accumulating in IO-treated populations, and ctDNA-directed adjuvant strategies are being evaluated prospectively in biomarker analysis of ongoing perioperative trials [51]. These early but promising biomarker-driven treatment strategies have important challenges that remain to be addressed, including sensitivity in non-metastatic disease, assay standardization, and a lack of prospective data demonstrating that ctDNA-guided treatment decisions improve oncologic outcomes compared with current standards of care. Today, ctDNA should be regarded as a powerful exploratory tool rather than a validated surrogate for routine treatment decisions in stage II disease.

## 5. Surgical Considerations

Across contemporary series, surgery remains the cornerstone of curative-intent therapy for stage II NSCLC, with lobectomy currently preferred for optimal oncologic control when anatomically feasible [53,57,58]. Large retrospective analyses over the past 20 years have consistently shown that most clinical stage II patients still undergo upfront resection, but fewer than half of those go on to receive indicated adjuvant therapy [59]. As systemic therapies improve and are associated with significant OS improvements, this underutilization is problematic and especially critical in patients pathologically upstaged at resection. There is an increased need for systems to ensure adherence to intended oncologic therapy after resection or to shift toward neoadjuvant or perioperative protocols when adherence is uncertain [60,61].

A significant concern with the shift to induction therapy for stage II patients is the potential for surgical attrition. Across trials, surgical attrition with neoadjuvant chemo-IO was 7–22% and was comparable to that in the control arms with neoadjuvant chemotherapy alone [37]. Upfront surgery technically has a 0% preoperative attrition rate, but if the priority is ensuring patients receive intended local and systemic therapies, neoadjuvant approaches may better facilitate this.

Beyond attrition, a complete risk–benefit assessment of perioperative chemo-IO must incorporate immune-related adverse events (irAEs) and toxicity. Grade 3–4 irAEs—most commonly pneumonitis, hepatitis, and colitis—occur in approximately 10–20% of patients receiving perioperative IO but resulted in the surgical attrition of 1–3% of patients across most neoadjuvant and perioperative trials [21,22,25]. For the surgeon, pneumonitis is particularly important as it may delay planned resection and is radiologically difficult to distinguish from postoperative pulmonary complications [21,22,25]. Additionally, discontinuation of adjuvant IO due to systemic toxicity occurred in 18–20% of patients in the adjuvant-only trials [10,11,12,13]. Perioperative IO should be used with particular caution in patients with active autoimmune conditions, impaired pulmonary reserve, or poor performance status—populations that were largely excluded from landmark trials. Patients harboring driver mutations should not receive IO-based regimens as the primary neoadjuvant strategy, given inferior IO response rates and elevated risk of irAEs when TKIs are subsequently administered [52].

A common concern with chemo-IO is the potential for hilar and mediastinal fibrosis, making operative dissection more difficult. Surgeons should anticipate the potential for more challenging hilar dissection, especially if there was clinically evident hilar nodal disease and a significant response to the induction therapy [62]. Nevertheless, the data show that perioperative chemo-IO is surgically feasible, with R0 rates exceeding 80–90% [23,37]. Similarly, minimally invasive surgery remains feasible, with roughly 30% to 50% of cases performed via video-assisted or robotic-assisted thoracoscopic surgery, and conversion rates remaining stable compared with the chemotherapy-alone era [63,64]. Similarly, there is a trend toward fewer pneumonectomies in the IO arms, likely due to improved tumor response with the addition of IO.

Systemic therapy trials for stage II NSCLC primarily focus on oncologic outcomes. Interestingly, novel trials are being conducted through a surgical lens, with primary endpoints focused on resection rates. The multicenter, phase II MDT-BRIDGE study does so by investigating a bridging strategy for patients with resectable or borderline-resectable stage IIB-IIIB NSCLC [31]. A multidisciplinary team assesses patients prior to starting therapy and then reassesses surgical eligibility after two cycles of chemo-IO, transitioning patients to either surgery or definitive chemoradiotherapy. Results from a pre-planned efficacy analysis presented at ESMO 2025 met the primary objective, demonstrating a 95% resection rate in the initial resectable cohort and an 82% surgical conversion rate among those previously considered borderline-resectable [31]. Fifteen percent of patients enrolled in this trial have stage II disease [31]. These data highlight the role of neoadjuvant chemo-IO in downstaging cancer and increasing the possibility of curative-intent surgery for many patients who previously were relegated to a non-operative approach.

## 6. Conclusions

The shift to perioperative IO has transformed stage II NSCLC from a purely surgical issue into a complex, multidisciplinary management challenge. Although technical challenges like immune-mediated fibrosis exist, they do not outweigh the survival advantages of early systemic treatment. The surgeon’s role has shifted from being primarily an interventionist to being part of a coordinated team in which molecular profiling, agent selection, and timing are just as critical as the resection itself.

Recent years have demonstrated a significant paradigm shift in the treatment landscape for clinical stage II NSCLC, resulting in a new standard of care. Treatment modalities are constantly evolving; in NSCLC, the process will only continue to become more personalized and multidisciplinary. As such, it is crucial that all patients undergo biomarker testing at the time of diagnosis to appropriately guide therapy. For tumors without actionable drivers, neoadjuvant and perioperative chemo-IO provides meaningful improvements in pathologic response, DFS, and OS. For those with known driver mutations (e.g., EGFR, ALK), adjuvant TKI is preferred to IO, with neoadjuvant TKI strategies actively being studied. Surgery—typically lobectomy with mediastinal lymph node dissection—remains central to curative-intent therapy in stage II disease but must be carefully integrated with systemic treatment. Ongoing questions about sequencing, treatment duration, patient selection, and the integration of MRD, ctDNA, and novel agents define current research priorities. Until comparative data are available, the optimal treatment of stage II NSCLC should be personalized, combining trial-based evidence with patient comorbidities, molecular profiles, and local multidisciplinary expertise.

## 7. Future Directions

Recent trials using IO and TKIs in resectable NSCLC have had incredible results with significant survival benefit and dramatically altered the care of resectable stage II disease. Currently ongoing trials seek to improve upon these results. For patients with EGFR mutations and ALK translocations, trials now explore introducing those agents earlier and better defining the optimal length of treatment. There are trials exploring additional actionable targeted agents in resectable disease. These trials accrue slowly due to low numbers of patients with each mutation, but umbrella trial designs are helping to improve accrual and keep trials open. For patients without targetable mutations, trials are exploring how to improve pCR rates with combinations that include ADCs and dual IO. We also now use the response to neoadjuvant chemo-IO to direct eligibility to ongoing trials and further personalize care. In patients with a pCR, trials explore de-escalating care and, in those with residual viable tumors, care escalation with novel agents including tumor specific mRNA vaccines is under investigation. MRD is explored in all of these settings with the hope that it can soon be incorporated into multidisciplinary care.

## Figures and Tables

**Table 1:** Landmark studies and trials involving immunotherapy as a treatment modality in resectable NSCLC.

Trial Name	Publication Year(s)	N	IO Agent	Setting	DFS/EFS	OS	Other
IMpower010 [10,11,12]	2021 2023 2025	1280	Atezolizumab	Adjuvant	5Y HR 0.66 (0.50–0.88)	5Y HR 0.71 (0.49–1.03)	NR
PEARLS/KEYNOTE -091 [13]	2022	1177	Pembrolizumab	Adjuvant	4Y HR 0.76 (0.63–0.91)	4Y HR 0.87 (0.67–1.15)	NR
CheckMate 159 [14,15,16]	2018 2019 2023	21	Nivolumab	Neoadjuvant	5Y HR 0.70 (0.39–1.24)	5Y HR 0.81 (0.42–1.56)	MPR 45% pCR~15% 95.2% R0 Resection
LCMC3 [17,18]	2022 2023	181	Atezolizumab	Neoadjuvant/Perioperative	3Y HR 0.30 (0.09–0.97)	3Y HR 0.14 (0.02–1.06)	MPR 20% pCR 7% 91–92% R0 Resection
CheckMate 816 [19,20]	2022 2025	358	Nivolumab	Neoadjuvant	5Y HR 0.68 (0.51–0.91)	5Y HR 0.72 (0.52–0.99)	MPR 37% pCR 24% 80–83% R0 Resection
KEYNOTE-671 [13,21,22,23,24,34]	2022 2023 2024 2026	797	Pembrolizumab	Perioperative	5Y HR 0.58 (0.48–0.69)	5Y HR 0.74 (0.69–0.92)	MPR 30% pCR 18% 92% R0 Resection
AEGEAN [25,26]	2023 2025	802	Durvalumab	Perioperative	3Y HR 0.68 (0.53–0.88)	3Y HR 0.71 (0.52–0.97)	MPR 33% pCR 17% 94.7% R0 Resection
CheckMate 77T [27]	2024	461	Nivolumab	Perioperative	18mo HR 0.58 (0.43–0.78)	18mo HR 0.85 (0.61–1.18)	MPR 35% pCR 25% 88–89% R0 Resection
NEOTORCH [28]	2024	401	Toripalimab	Perioperative	3Y HR 0.40 (0.28–0.57)	3Y HR 0.62 (0.38–0.99)	MPR 48.5% pCR 24.8%
NeoCOAST [29]	2023	84	Durvalumab	Neoadjuvant	NR	NR	MPR 33.3% pCR 17%
NeoCOAST-2 [30]	2025	202	Durvalumab	Perioperative	NR	NR	MPR 63% vs. 50% vs. 42% pCR 35.2% vs. 25.7% vs. 20.3%
MDT-Bridge [31]	2024	140 Target	Durvalumab	Neoadjuvant	NR	NR	82% of borderline patients became resectable after induction, high nodal clearance
RATIONALE-315 [32,33]	2025 2026	453	Tislelizumab	Perioperative	3Y HR 0.56 (0.40–0.79)	3Y HR 0.62 (0.39–0.98)	MPR 56.1% pCR 40.7%

Abbreviations: DFS, disease-free survival; EFS, event-free survival; HR, hazard ratio; IO, immunotherapy; NR, not reported; Y, year; mo, month; MPR, major pathologic response; OS, overall survival; pCR, pathologic complete response. Hazard ratios are expressed as HR (95% confidence interval).

**Table 3:** Landmark studies and trials involving targeted therapy agents in resectable NSCLC.

Trial Name	Publication Year	N	Driver Mutation	Included Stages	Setting	Agent	DFS/EFS	OS	Other
ADAURA [39,40,41,42]	2020 2023 2025	682	EGFR	IB-IIIA	Adjuvant	Osimertinib	5Y HR 0.23 (0.18–0.30)	5Y HR 0.49 (0.33–0.73)	100% R0 Resection
NeoADAURA [43]	2025	358	EGFR	II-IIIB	Neoadjuvant	Osimertinib	1Y HR 0.50 (0.17–1.41) 1Y HR 0.73 (0.40–1.35	NR	MPR 25–26%
ALINA [44]	2024	257	ALK	IB-IIIA	Adjuvant	Alectinib	4Y HR 0.24 (0.13–0.43)	NR	100% R0 Resection
ELEVATE [45]	2025	100	ALK	IB-IIIB	Adjuvant	Ensartinib	2Y HR 0.20 (0.09–0.31)	NR	NR
ALNEO [46,47]	2021 2025	33	ALK	III	Perioperative	Alectinib	NR	NR	MPR 46% pCR 12% 86% R0 Resection
NAUTIKA1 [48]	2022	5	Several	IB-IIIB	Perioperative	Alectinib	NR	NR	MPR 61% pCR 25% 93% R0 Resection

Abbreviations: ALK, anaplastic lymphoma kinase; DFS, disease-free survival; EGFR, epidermal growth factor receptor; HR, hazard ratio; MPR, major pathologic response; NR, not reported; OS, overall survival; pCR, pathologic complete response; Y, year. Hazard ratios are expressed as HRs (95% confidence interval).

## Data Availability

No new data were analyzed for the purposes of this study.

## Abbreviations

The following abbreviations are used in this manuscript:

NSCLC	non-small-cell lung cancer
IO	immunotherapy
PD-L1	programmed death-ligand 1
DFS	disease-free survival
HR	hazard ratio
OS	overall survival
EFS	event-free survival
pCR	pathologic complete response
MPR	major pathologic response
ADC	antibody–drug conjugate
TKI	tyrosine kinase inhibitor
EGFR	epidermal growth factor receptor
ALK	anaplastic lymphoma kinase
irAE	immune-related adverse event
ctDNA	circulating tumor DNA
MRD	molecular residual disease

## References

[1] Siegel R.L., Giaquinto A.N., Jemal A. (2024). Cancer Statistics, 2024. CA A Cancer J. Clin..

[2] Rami-Porta R., Nishimura K.K., Giroux D.J., Detterbeck F., Cardillo G., Edwards J.G., Fong K.M., Giuliani M., Huang J., Kernstine K.H. (2024). The International Association for the Study of Lung Cancer Lung Cancer Staging Project: Proposals for Revision of the TNM Stage Groups in the Forthcoming (Ninth) Edition of the TNM Classification for Lung Cancer. J. Thorac. Oncol..

[3] Goldstraw P., Crowley J., Chansky K., Giroux D.J., Groome P.A., Rami-Porta R., Postmus P.E., Rusch V., Sobin L. (2007). The IASLC Lung Cancer Staging Project: Proposals for the Revision of the TNM Stage Groupings in the Forthcoming (Seventh) Edition of the TNM Classification of Malignant Tumours. J. Thorac. Oncol..

[4] Arriagada R., Bergman B., Dunant A., Le Chevalier T., Pignon J.P., Vansteenkiste J., International Adjuvant Lung Cancer Trial Collaborative Group (2004). Cisplatin-Based Adjuvant Chemotherapy in Patients with Completely Resected Non–Small-Cell Lung Cancer. N. Engl. J. Med..

[5] Winton T., Livingston R., Johnson D., Rigas J., Johnston M., Butts C., Cormier Y., Goss G., Inculet R., Vallieres E. (2005). Vinorelbine plus Cisplatin vs. Observation in Resected Non–Small-Cell Lung Cancer. N. Engl. J. Med..

[6] Douillard J.-Y., Rosell R., De Lena M., Carpagnano F., Ramlau R., Gonzáles-Larriba J.L., Grodzki T., Pereira J.R., Le Groumellec A., Lorusso V. (2006). Adjuvant Vinorelbine plus Cisplatin versus Observation in Patients with Completely Resected Stage IB–IIIA Non-Small-Cell Lung Cancer (Adjuvant Navelbine International Trialist Association [ANITA]): A Randomised Controlled Trial. Lancet Oncol..

[7] Pignon J.-P., Tribodet H., Scagliotti G.V., Douillard J.-Y., Shepherd F.A., Stephens R.J., Dunant A., Torri V., Rosell R., Seymour L. (2008). Lung Adjuvant Cisplatin Evaluation: A Pooled Analysis by the LACE Collaborative Group. JCO.

[8] Zhang R., Zou C., Zeng L., Zhang Y. (2025). Perioperative Immunotherapy in Nonsmall Cell Lung Cancer. Curr. Opin. Oncol..

[9] Muthusamy B., Patil P.D., Pennell N.A. (2022). Perioperative Systemic Therapy for Resectable Non–Small Cell Lung Cancer. J. Natl. Compr. Cancer Netw..

[10] Felip E., Altorki N., Zhou C., Csőszi T., Vynnychenko I., Goloborodko O., Luft A., Akopov A., Martinez-Marti A., Kenmotsu H. (2021). Adjuvant Atezolizumab after Adjuvant Chemotherapy in Resected Stage IB–IIIA Non-Small-Cell Lung Cancer (IMpower010): A Randomised, Multicentre, Open-Label, Phase 3 Trial. Lancet.

[11] Felip E., Altorki N., Zhou C., Vallières E., Martínez-Martí A., Rittmeyer A., Chella A., Reck M., Goloborodko O., Huang M. (2023). Overall Survival with Adjuvant Atezolizumab after Chemotherapy in Resected Stage II-IIIA Non-Small-Cell Lung Cancer (IMpower010): A Randomised, Multicentre, Open-Label, Phase III Trial. Ann. Oncol..

[12] Felip E., Altorki N., Zhou C., Vallières E., Csoszi T., Vynnychenko I.O., Goloborodko O., Rittmeyer A., Reck M., Martinez-Marti A. (2025). Five-Year Survival Outcomes With Atezolizumab After Chemotherapy in Resected Stage IB-IIIA Non–Small Cell Lung Cancer (IMpower010): An Open-Label, Randomized, Phase III Trial. JCO.

[13] O’Brien M., Paz-Ares L., Marreaud S., Dafni U., Oselin K., Havel L., Esteban E., Isla D., Martinez-Marti A., Faehling M. (2022). Pembrolizumab versus Placebo as Adjuvant Therapy for Completely Resected Stage IB–IIIA Non-Small-Cell Lung Cancer (PEARLS/KEYNOTE-091): An Interim Analysis of a Randomised, Triple-Blind, Phase 3 Trial. Lancet Oncol..

[14] Forde P.M., Chaft J.E., Smith K.N., Anagnostou V., Cottrell T.R., Hellmann M.D., Zahurak M., Yang S.C., Jones D.R., Broderick S. (2018). Neoadjuvant PD-1 Blockade in Resectable Lung Cancer. N. Engl. J. Med..

[15] Bott M.J., Yang S.C., Park B.J., Adusumilli P.S., Rusch V.W., Isbell J.M., Downey R.J., Brahmer J.R., Battafarano R., Bush E. (2019). Initial Results of Pulmonary Resection after Neoadjuvant Nivolumab in Patients with Resectable Non–Small Cell Lung Cancer. J. Thorac. Cardiovasc. Surg..

[16] Rosner S., Reuss J.E., Zahurak M., Zhang J., Zeng Z., Taube J., Anagnostou V., Smith K.N., Riemer J., Illei P.B. (2023). Five-Year Clinical Outcomes after Neoadjuvant Nivolumab in Resectable Non-Small Cell Lung Cancer. Clin. Cancer Res..

[17] Chaft J.E., Oezkan F., Kris M.G., Bunn P.A., Wistuba I.I., Kwiatkowski D.J., Owen D.H., Tang Y., Johnson B.E., Lee J.M. (2022). Neoadjuvant Atezolizumab for Resectable Non-Small Cell Lung Cancer: An Open-Label, Single-Arm Phase II Trial. Nat. Med..

[18] Rusch V.W., Nicholas A., Patterson G.A., Waqar S.N., Toloza E.M., Haura E.B., Raz D.J., Reckamp K.L., Merritt R.E., Owen D.H. (2023). Surgical Results of the Lung Cancer Mutation Consortium 3 Trial: A Phase II Multicenter Single-Arm Study to Investigate the Efficacy and Safety of Atezolizumab as Neoadjuvant Therapy in Patients with Stages IB-Select IIIB Resectable Non–Small Cell Lung Cancer. J. Thorac. Cardiovasc. Surg..

[19] Forde P.M., Spicer J., Lu S., Provencio M., Mitsudomi T., Awad M.M., Felip E., Broderick S.R., Brahmer J.R., Swanson S.J. (2022). Neoadjuvant Nivolumab plus Chemotherapy in Resectable Lung Cancer. N. Engl. J. Med..

[20] Forde P.M., Spicer J.D., Provencio M., Mitsudomi T., Awad M.M., Wang C., Lu S., Felip E., Swanson S.J., Brahmer J.R. (2025). Overall Survival with Neoadjuvant Nivolumab plus Chemotherapy in Lung Cancer. N. Engl. J. Med..

[21] Wakelee H., Liberman M., Kato T., Tsuboi M., Lee S.-H., Gao S., Chen K.-N., Dooms C., Majem M., Eigendorff E. (2023). Perioperative Pembrolizumab for Early-Stage Non–Small-Cell Lung Cancer. N. Engl. J. Med..

[22] Spicer J.D., Garassino M.C., Wakelee H., Liberman M., Kato T., Tsuboi M., Lee S.-H., Chen K.-N., Dooms C., Majem M. (2024). Neoadjuvant Pembrolizumab plus Chemotherapy Followed by Adjuvant Pembrolizumab Compared with Neoadjuvant Chemotherapy Alone in Patients with Early-Stage Non-Small-Cell Lung Cancer (KEYNOTE-671): A Randomised, Double-Blind, Placebo-Controlled, Phase 3 Trial. Lancet.

[23] Liberman M., Jones D.R., Wakelee H., Gao S., Halmos B., Nadal E., Łowczak A., Reck M., Novello S., Matias D. (2026). Clinical Characteristics and Surgical Outcomes of Patients Receiving Perioperative Pembrolizumab in KEYNOTE-671. Ann. Thorac. Surg..

[24] Tsuboi M., Wakelee H., Garassino M.C., Gao S., Luft A., Chen K.-N., Spicer J.D., Zhu Y., Saji H., Okada M. (2026). A Subgroup Analysis of Perioperative Pembrolizumab in Clinical Stage II Non-Small-Cell Lung Cancer from the Randomized KEYNOTE-671 Study. Eur. J. Cardio-Thorac. Surg..

[25] Heymach J.V., Harpole D., Mitsudomi T., Taube J.M., Galffy G., Hochmair M., Winder T., Zukov R., Garbaos G., Gao S. (2023). Perioperative Durvalumab for Resectable Non–Small-Cell Lung Cancer. N. Engl. J. Med..

[26] Mitsudomi T., Heymach J.V., Reck M., Taube J.M., Gao S., Horio Y., You J., Li G., Van Luong D., Saeteng S. (2025). Surgical Outcomes with Neoadjuvant Durvalumab Plus Chemotherapy Followed by Adjuvant Durvalumab in Resectable NSCLC. J. Thorac. Oncol..

[27] Cascone T., Awad M.M., Spicer J.D., He J., Lu S., Sepesi B., Tanaka F., Taube J.M., Cornelissen R., Havel L. (2024). Perioperative Nivolumab in Resectable Lung Cancer. N. Engl. J. Med..

[28] Lu S., Zhang W., Wu L., Wang W., Zhang P., Fang W., Xing W., Chen Q., Yang L., Neotorch Investigators (2024). Perioperative Toripalimab Plus Chemotherapy for Patients with Resectable Non–Small Cell Lung Cancer: The Neotorch Randomized Clinical Trial. JAMA.

[29] Cascone T., Kar G., Spicer J.D., García-Campelo R., Weder W., Daniel D.B., Spigel D.R., Hussein M., Mazieres J., Oliveira J. (2023). Neoadjuvant Durvalumab Alone or Combined with Novel Immuno-Oncology Agents in Resectable Lung Cancer: The Phase II NeoCOAST Platform Trial. Cancer Discov..

[30] Cascone T., Bonanno L., Guisier F., Insa A., Liberman M., Bylicki O., Livi L., Egenod T., Corre R., Kim D.-W. (2025). Perioperative Durvalumab plus Chemotherapy plus New Agents for Resectable Non-Small-Cell Lung Cancer: The Platform Phase 2 NeoCOAST-2 Trial. Nat. Med..

[31] Reck M., Nadal E., Girard N., Filippi A.R., Martin L.W., Gay C.M., Petersen C., Gale D., Emeribe U.A., Georgoulia N. (2024). MDT-BRIDGE: Neoadjuvant Durvalumab Plus Chemotherapy Followed by Either Surgery and Adjuvant Durvalumab or Chemoradiotherapy and Consolidation Durvalumab in Resectable or Borderline-Resectable Stage IIB–IIIB NSCLC. Clin. Lung Cancer.

[32] Yue D., Wang W., Liu H., Chen Q., Chen C., Liu L., Zhang P., Zhao G., Yang F., Han G. (2025). Perioperative Tislelizumab plus Neoadjuvant Chemotherapy for Patients with Resectable Non-Small-Cell Lung Cancer (RATIONALE-315): An Interim Analysis of a Randomised Clinical Trial. Lancet Respir. Med..

[33] Wang C., Wang W., Liu H., Chen Q., Chen C., Liu L., Zhang P., Zhao G., Yang F., Han G. (2026). Perioperative Tislelizumab plus Neoadjuvant Chemotherapy for Patients with Resectable Non-Small-Cell Lung Cancer: Final Analysis of the Randomized RATIONALE-315 Trial. Ann. Oncol..

[34] Wakelee H., Spicer J., Gao S., Liberman M., Tsuboi M., Kato T., Chen K.-N., Dooms C., Majem M., Martinengo G.L. (2025). LBA67 Perioperative Pembrolizumab in Early-Stage Non-Small- Cell Lung Cancer (NSCLC): 5-Year Follow-up from KEYNOTE- 671. Ann. Oncol..

[35] Saw S.P.L., Ong B.-H., Chua K.L.M., Takano A., Tan D.S.W. (2021). Revisiting Neoadjuvant Therapy in Non-Small-Cell Lung Cancer. Lancet Oncol..

[36] Aburaki R., Fujiwara Y., Chida K., Horita N., Nagasaka M. (2024). Surgical and Safety Outcomes in Patients with Non-Small Cell Lung Cancer Receiving Neoadjuvant Chemoimmunotherapy versus Chemotherapy Alone: A Systematic Review and Meta-Analysis. Cancer Treat. Rev..

[37] Sorin M., Prosty C., Ghaleb L., Nie K., Katergi K., Shahzad M.H., Dubé L.-R., Atallah A., Swaby A., Dankner M. (2024). Neoadjuvant Chemoimmunotherapy for NSCLC: A Systematic Review and Meta-Analysis. JAMA Oncol..

[38] Rossi G., Barcellini L., Tagliamento M., Tanda E.T., Garassino M.C., Blondeaux E., Delucchi V., Spagnolo F., Del Mastro L., Genova C. (2025). Immunotherapy for Resectable NSCLC: Neoadjuvant/Perioperative Followed by Surgery over Surgery Followed by Adjuvant. Systematic Review and Meta-Analysis with Subgroup Analyses. ESMO Open.

[39] Wu Y.-L., Tsuboi M., He J., John T., Grohe C., Majem M., Goldman J.W., Laktionov K., Kim S.-W., Kato T. (2020). Osimertinib in Resected *EGFR* -Mutated Non–Small-Cell Lung Cancer. N. Engl. J. Med..

[40] Tsuboi M., Herbst R.S., John T., Kato T., Majem M., Grohé C., Wang J., Goldman J.W., Lu S., Su W.-C. (2023). Overall Survival with Osimertinib in Resected *EGFR* -Mutated NSCLC. N. Engl. J. Med..

[41] Herbst R.S., Wu Y.-L., John T., Grohe C., Majem M., Wang J., Kato T., Goldman J.W., Laktionov K., Kim S.-W. (2023). Adjuvant Osimertinib for Resected EGFR-Mutated Stage IB-IIIA Non–Small-Cell Lung Cancer: Updated Results From the Phase III Randomized ADAURA Trial. JCO.

[42] Herbst R.S., John T., Grohé C., Goldman J.W., Kato T., Laktionov K., Bonanno L., Tiseo M., Majem M., Dómine M. (2025). Molecular Residual Disease Analysis of Adjuvant Osimertinib in Resected EGFR-Mutated Stage IB–IIIA Non-Small-Cell Lung Cancer. Nat. Med..

[43] He J., Tsuboi M., Weder W., Chen K.-N., Hochmair M.J., Shih J.-Y., Lee S.Y., Lee K.-Y., Nhung N.V., Saeteng S. (2025). Neoadjuvant Osimertinib for Resectable *EGFR* -Mutated Non–Small Cell Lung Cancer. JCO.

[44] Wu Y.-L., Dziadziuszko R., Ahn J.S., Barlesi F., Nishio M., Lee D.H., Lee J.-S., Zhong W., Horinouchi H., Mao W. (2024). Alectinib in Resected *ALK* -Positive Non–Small-Cell Lung Cancer. N. Engl. J. Med..

[45] ESMO Congress 2025—Conference Calendar—ESMO Congress 2025. https://cslide.ctimeetingtech.com/esmo2025/attendee/confcal/show/session/153.

[46] Leonetti A., Minari R., Boni L., Gnetti L., Verzè M., Ventura L., Musini L., Tognetto M., Tiseo M. (2021). Phase II, Open-Label, Single-Arm, Multicenter Study to Assess the Activity and Safety of Alectinib as Neoadjuvant Treatment in Surgically Resectable Stage III ALK-Positive NSCLC: ALNEO Trial. Clin. Lung Cancer.

[47] Leonetti A., Boni L., Gnetti L., Cortinovis D.L., Pasello G., Mazzoni F., Bearz A., Gelsomino F., Passiglia F., Pilotto S. (2025). Alectinib as Neoadjuvant Treatment in Potentially Resectable Stage III ALK-Positive NSCLC: Final Analysis of ALNEO Phase II Trial (GOIRC-01-2020-ML42316). JCO.

[48] Lee J.M., Sepesi B., Toloza E.M., Lin J., Pass H.I., Johnson B.E., Heymach J.V., Johnson M.L., Ding B., Schulze K. (2022). EP02.04-005 Phase II NAUTIKA1 Study of Targeted Therapies in Stage II-III NSCLC: Preliminary Data of Neoadjuvant Alectinib for ALK+ NSCLC. J. Thorac. Oncol..

[49] Zhou Y., Li A., Yu H., Wang Y., Zhang X., Qiu H., Du W., Luo L., Fu S., Zhang L. (2024). Neoadjuvant-Adjuvant vs Neoadjuvant-Only PD-1 and PD-L1 Inhibitors for Patients with Resectable NSCLC: An Indirect Meta-Analysis. JAMA Netw. Open.

[50] Patel S.P., Othus M., Chen Y., Wright G.P., Yost K.J., Hyngstrom J.R., Hu-Lieskovan S., Lao C.D., Fecher L.A., Truong T.-G. (2023). Neoadjuvant–Adjuvant or Adjuvant-Only Pembrolizumab in Advanced Melanoma. N. Engl. J. Med..

[51] Booth A., Hillman S., Laumann K., Zhao Y.-Q., LeBlanc M., Chansky K., Giri S., Dill J., Johnson J., Kelley K. (2026). PROSPECT-LUNG: A National Clinical Trials Network Trial Advancing Pragmatic Innovation in Cancer Clinical Trials. J. Thorac. Oncol..

[52] Schoenfeld A.J., Arbour K.C., Rizvi H., Iqbal A.N., Gadgeel S.M., Girshman J., Kris M.G., Riely G.J., Yu H.A., Hellmann M.D. (2019). Severe Immune-Related Adverse Events Are Common with Sequential PD-(L)1 Blockade and Osimertinib. Ann. Oncol..

[53] Kim S.S., Cooke D.T., Kidane B., Tapias L.F., Lazar J.F., Awori Hayanga J.W., Patel J.D., Neal J.W., Abazeed M.E., Willers H. (2025). The Society of Thoracic Surgeons Expert Consensus on the Multidisciplinary Management and Resectability of Locally Advanced Non-Small Cell Lung Cancer. Ann. Thorac. Surg..

[54] Liu S.-Y., Zhang J.-T., Zeng K.-H., Wu Y.-L. (2022). Perioperative Targeted Therapy for Oncogene-Driven NSCLC. Lung Cancer.

[55] Woodard G.A., Kramer R.J., Blakely C.M. (2026). Neoadjuvant Tyrosine Kinase Inhibitors. Thorac. Surg. Clin..

[56] Occhipinti M., Imbimbo M., Ferrara R., Simeon V., Fiscon G., Marchal C., Skoetz N., Viscardi G. (2025). Adjuvant Epidermal Growth Factor Receptor (EGFR) Tyrosine Kinase Inhibitors (TKIs) for the Treatment of People with Resected Stage I to III Non-Small-Cell Lung Cancer and EGFR Mutation. Cochrane Database Syst. Rev..

[57] Howington J., Souter L.H., Arenberg D., Blasberg J., Detterbeck F., Farjah F., Lanuti M., Leighl N., Videtic G.M., Murthy S. (2025). Management of Patients with Early-Stage Non-Small Cell Lung Cancer. Chest.

[58] Kidane B., Bott M., Spicer J., Backhus L., Chaft J., Chudgar N., Colson Y., D’Amico T.A., David E., Lee J. (2023). The American Association for Thoracic Surgery (AATS) 2023 Expert Consensus Document: Staging and Multidisciplinary Management of Patients with Early-Stage Non–Small Cell Lung Cancer. J. Thorac. Cardiovasc. Surg..

[59] Kehl K.L., Zahrieh D., Yang P., Hillman S.L., Tan A.D., Sands J.M., Oxnard G.R., Gillaspie E.A., Wigle D., Malik S. (2022). Rates of Guideline-Concordant Surgery and Adjuvant Chemotherapy Among Patients with Early-Stage Lung Cancer in the US ALCHEMIST Study (Alliance A151216). JAMA Oncol..

[60] Jindani R., Loh I., Rodriguez-Quintero J.H., Ha G., Rosario J., Cohen B., Nobel T.B., Vimolratana M., Chudgar N.P., Stiles B.M. (2025). Clinical Stage II Non-Small Cell Lung Cancer: Can We “Just Give Adjuvant Therapy”?. J. Thorac. Dis..

[61] Howington J.A., Blum M.G., Chang A.C., Balekian A.A., Murthy S.C. (2013). Treatment of Stage I and II Non-Small Cell Lung Cancer. Chest.

[62] Feldman H., Sepesi B., Leung C.H., Lin H., Weissferdt A., Pataer A., William W.N., Walsh G.L., Rice D.C., Roth J.A. (2024). Surgical Outcomes after Chemotherapy plus Nivolumab and Chemotherapy plus Nivolumab and Ipilimumab in Patients with Non–Small Cell Lung Cancer. J. Thorac. Cardiovasc. Surg..

[63] Kneuertz P.J., Villamizar N., Altorki N.K., Phillips J.D., Schnorr P., Jones D., Scott S., D’Souza D.M., Baiu I., Abdel-Rasoul M. (2025). Minimally Invasive Resection of Non–Small Cell Lung Cancer after Chemoimmunotherapy: A Multicenter Study in Academic Hospitals. J. Thorac. Cardiovasc. Surg..

[64] Pohlman A., Marten A., Coughlin J.M., Lubawski J., Raad W., Abdelsattar Z.M. (2026). Impact of Neoadjuvant Therapy, Tumor Size and Surgical Approach on Conversion to Open Thoracotomy During Lobectomy. Ann. Thorac. Surg..

